# Vulvodynia: a neuroinflammatory pain syndrome originating in pelvic visceral nerve plexuses due to mechanical factors

**DOI:** 10.1007/s00404-022-06424-4

**Published:** 2022-02-11

**Authors:** Jacob Bornstein, Eilam Palzur, Michael Swash, Peter Petros

**Affiliations:** 1 Pain Research Laboratory, Research institute, Galilee Medical Center and Azrieli Faculty of Medicine, Bar Ilan University, 89 Road, 22100 Nahariya, Israel; 2 Barts and the London School of Medicine, QMUL, London, E1 2AD UK; 3 Catedratico Convidado da Disciplina de Neurologia da Faculdade de Medicina de Lisboa (Translational Physiology), University of Lisbon, Lisbon, Portugal; 4 School of Mechanical and Mathematical Engineering, University of Western Australia, 35 Stirling Highway, Perth, WA 6009 Australia

**Keywords:** Neuroproliferation, Provoked vulvodynia, Uterosacral ligaments

## Abstract

This short opinion aimed to present the evidence to support our hypothesis that vulvodynia is a neuroinflammatory pain syndrome originating in the pelvic visceral nerve plexuses caused by the failure of weakened uterosacral ligaments (USLs) to support the pelvic visceral nerve plexuses, i.e., T11–L2 sympathetic and S2–4 parasympathetic plexuses. These are supported by the USLs, 2 cm from their insertion to the cervix. They innervate the pelvic organs, glands, and muscles. If the USLs are weak or lax, gravitational force or even the muscles may distort and stimulate the unsupported plexuses. Inappropriate afferent signals could then be interpreted as originating from an end-organ site. Activation of sensory visceral nerves causes a neuro-inflammatory response in the affected tissues, leading to neuroproliferation of small peripheral sensory nerve fibers, which may cause hyperalgesia and allodynia in the territory of the damaged innervation. Repair of the primary abnormality of USL laxity, responsible for mechanical stimulation of the pelvic sensory plexus, may lead to resolution of the pain syndrome.

## Introduction

Vulvodynia, formerly termed “vulvar vestibulitis”, affects 8–10% of women of all ages, causing vulval pain and dyspareunia [1].

The cause of vulvodynia is unknown, and it consists of a complex multifactorial disorder with a “heterogenicity of causes” [2]. Hypotheses for associated factors, when there is no evident local cause, range from hormonal, genetic, and altered autoimmune responses to allergies, neuropathic changes, afferent C-fiber activation, psychosomatic, psychiatric, increased urinary oxalates, and pelvic floor overactivity.

Provoked vulvodynia is characterized by extreme and usually symmetric sensitivity at the vestibule, close to the hymenal base and the clitoral area and paraurethral region. Quality of life can be significantly impaired. Bilateral pain symmetry, if present, is inconsistent with pudendal neuropathy. Inflammatory cells and neuroproliferative changes have been found on cutaneous histological examination [3]; however, the vagina and vulva usually show no redness or other overt inflammatory signs [4]. The severity of this pain often leads to anxiety, depression, poor quality of life, and deterioration of relationships, because the pain, often extreme, prohibits sexual intercourse. These manifestations, coupled with the absence of any conventional intrapelvic pathogenic findings, have suggested that vulval pain has a psychological basis. The pain is associated with local, vestibular hyperalgesia and allodynia (pain induced by a non-painful stimulus). In half of affected women, increased sensitivity is found only in the posterior half of the vestibule, whereas the others have both anterior and posterior allodynia.

**Fig. 1:**
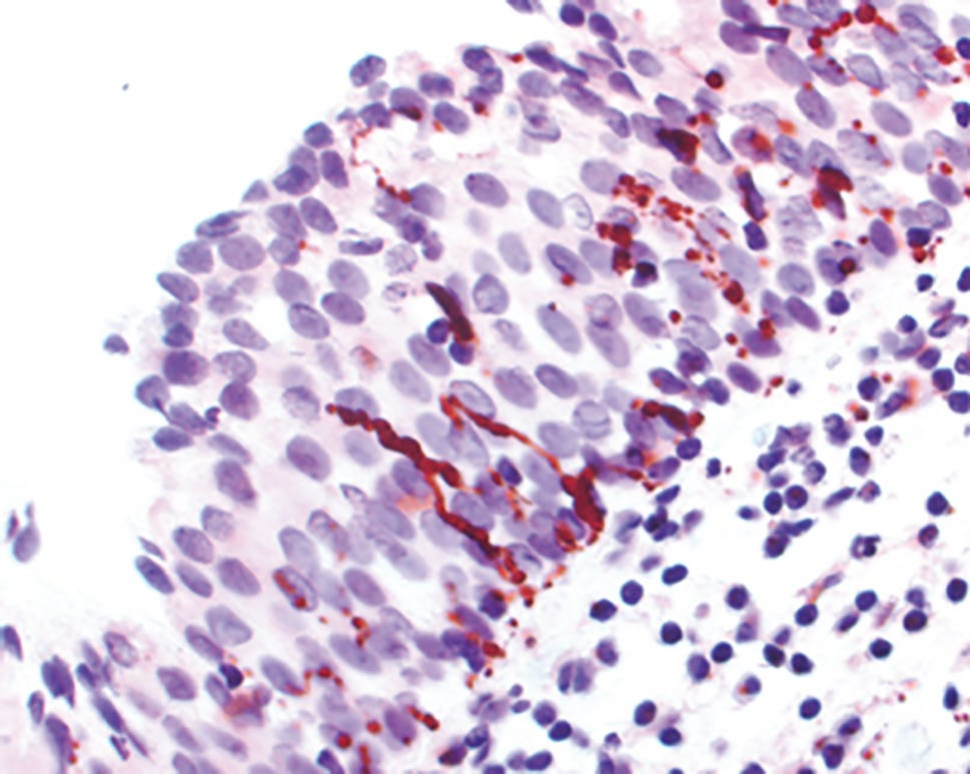
Neuroproliferation. Protein gene product (PGP) 9.5 stain in a tissue specimen from a woman with provoked vulvodynia showing nerve fibers intruding into the vulval epithelium to > 50% of its depth. Magnification: × 400

A peripheral neurologic mechanism leading to neuroproliferation (Fig. 1) has been recognized in women with vulvodynia and in animal models [3, 4]. However, the trigger for this mechanism remains unknown. Vulvodynia often co-occurs with other types of chronic pelvic pain of unknown origin (CPPU), for example, paraurethral, lower abdominal, sacral, and contact dyspareunia with perineal pain or burning [5]. CPPU, bladder symptoms of OAB, and abnormal emptying are components of the Posterior Fornix Syndrome* (6), which predictably co-occurs with chronic pelvic pain, urge, frequency, nocturia, and abnormal emptying, caused by uterosacral ligament laxity and cured or improved by repair thereof.

**Fig. 2:**
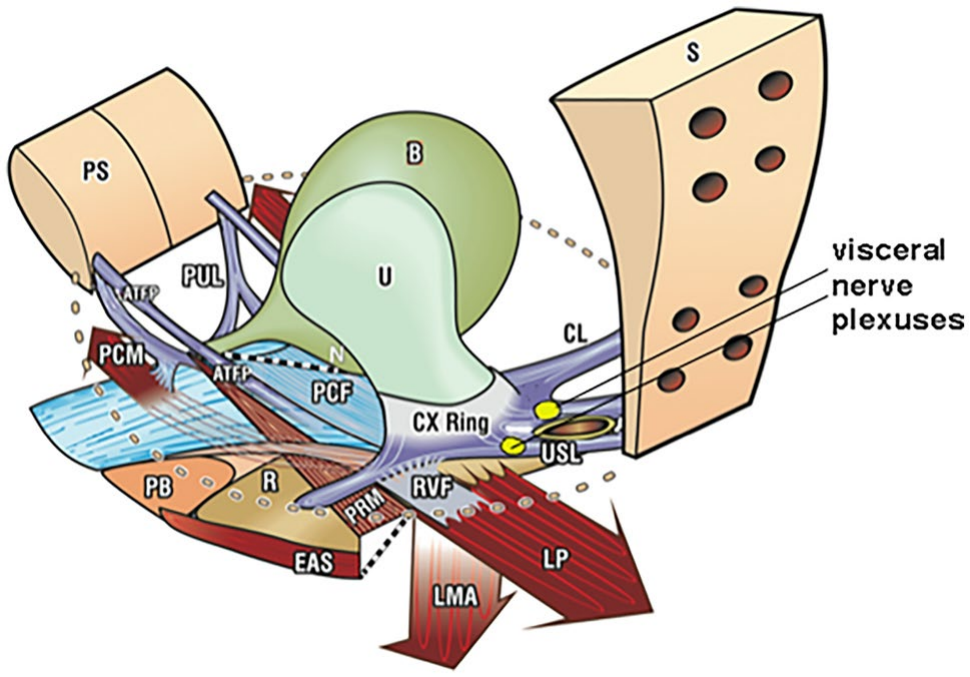
Opposite muscle forces (arrows) tension uterosacral ligaments (USL) to support nerve plexuses Organs *B* bladder, *R* rectum, *U* uterus, Bone: *PS* pubic symphysis, *S* sacrum, Suspensory ligaments: *PUL* pubourethral ligament, *ATFP* arcus tendineus fascia pelvis, *USL* uterosacral ligament, *CL* cardinal ligament, Muscle forces: *PCM* pubococcygeus muscle, *LP* levator plate, *LMA* longitudinal muscle of the anus, *PRM* puborectalis muscle. Supporting fascia: *PCF* pubocervical fascia, *RVF* rectovaginal fascia. Perineal anchoring structures: *PB* perineal body, *EAS* external anal sphincter

In a few cases, we have found that these pain syndromes can be improved or cured by uterosacral ligament (USL) repair [6], suggesting that the mechanism for CPPU relief is the restoration of intrapelvic ligamentous support for the T11–L2 and S2–4 visceral nerve plexuses (Fig. 2). We aimed to review the evidence for this causative relationship and emphasize its implications for patient management.

## Clinical evidence

We have reported that weakened USLs fail to support pelvic visceral neural plexuses, associated with vulvodynia. This concept is based on several clinical studies:

1. We have described three patients experiencing vulvodynia in whom vulval pain and co-occurring bladder symptoms were all cured by surgical reinforcement of the USLs [6].
2. In a subsequent randomized trial of 20 women with severe provoked vulvodynia, the vestibular allodynia could be temporarily alleviated by applying a large swab to the posterior vaginal fornix, thus supporting it [7]. Five patients experienced > 30% reduction in their pain levels, and the average reduction in pain with USL support using a 0–10 visual analogue scale was 18·4% ± 2·2%.
3. Ten women with provoked vulvodynia had local anesthetic injected into the USLs 2 cm from their insertion into the cervix (Bornstein Test) [9]. The pain disappeared from both sides of the vulva in eight women for 20 min and from one side of the vulva in two women [8]. The Bornstein Test was applied to another three women who had interstitial cystitis (IC) and multiple sites of chronic pelvic pain which were relieved partially or totally for 20 min following injection of local anesthetic [9]. Though with only three cases, this small experiment demonstrated a link between IC and vulvodynia.

## The uterosacral ligament hypothesis for vulvodynia causation

If the USLs are lax, gravitational force or contractile force from intrapelvic muscles may displace the unsupported plexuses, inadvertently stimulating them. Afferent signals then pass to the brain but are (wrongly) interpreted as coming from an end-organ site so that tenderness is experienced at the innervation target of the nerve. Activation of visceral sensory nerves induces the nervous system to cause a local neuro-inflammatory response to develop in the affected tissues, with macrophage, mast cell, and T-cell responses. This local neuro-inflammatory response exacerbates the pain; for example, nerve growth factors secreted from mast cell granules can induce neuroproliferation and sensitization. However, hyperalgesia and allodynia, and the neuro-inflammatory response will resolve if the primary abnormality, the USL laxity, is successfully repaired.

## Anatomical evidence behind the uterosacral ligament hypothesis

The T11–L2 and S2–4 visceral nerve plexuses are formed from sympathetic and parasympathetic nerve complexes. The sympathetic plexuses are derived from T11–12 cord segments (Frankenhauser’s plexus), and the parasympathetic innervations derived from S2–4 are located approximately 2 cm from the USL insertion into the posterior cervical ring. These merged plexuses innervate the pelvic organs, Bartholin glands, urethral glands, anal glands, and vulva. The visceral plexus is involved in modulating bladder and bowel continence and evacuation. The Frankenhauser’s plexuses are supported by the uterosacral ligaments (Fig. 2). The levator plate muscles and conjoint longitudinal muscle of the anus contract against the USLs, causing stretching and tension, providing mechanical support to these visceral nerve plexuses [10, 11].

Although our observations relate specifically to patients with vulvodynia, there are wider implications. For example, vulvodynia develops if the stimulated visceral nerve plexuses are those that innervate the Bartholin glands or vagina. If the afferent unmyelinated nerve fibers of the bladder, anal glands, urethra, or lower abdomen in the pelvic plexus are stimulated, the patient may develop interstitial cystitis, perianal pain, paraurethral pain, pelvic muscle spasm, abdominal pain, or coccydynia, which can all potentially be relieved by improving mechanical support to the pelvic floor.

## Cardinal and uterosacral ligament evidence

Somatic sensory nerve fibers innervate the vulva via the pudendal nerves (S2–4); however, allodynia in provoked vulvodynia is usually symmetrical. This is inconsistent with pudendal nerve involvement. Using neuropeptide and enzyme markers, Butler-Manuel et al. have demonstrated the presence of sympathetic, parasympathetic, nociceptive, and sensory-motor nerves in both cardinal ligaments and USLs, with sympathetic nerve ending density dominant in the lateral part of USLs [12]. Several sympathetic nerves were preganglionic, and their cell bodies lay within the pelvic plexus [8]. Butler-Manuel et al. have summarized, “The uterine supporting ligaments are a major pathway for autonomic nerves to the pelvic organs” [12]. These findings are supported by the demonstration of both myelinated and unmyelinated nerve fibers in the USLs [5, 12].

## Vestibular changes

Several studies have reported that hyperinnervation and nerve sprouting occurs in the vestibular stroma of the vestibule in women with vulvodynia (Fig. 1) [13, 14]. Immunohistochemically stained sections showed that the nerve fiber density in the stroma of women with vulvodynia was ten times greater than that of women in the control group [13]. Nociceptor nerve fibers penetrated the basal membrane and continued vertically, approaching the epithelial surface [14]. Increased vanilloid receptor VR1 (TR1) density was associated with allodynia [15, 16], and increased numbers of T cells, macrophages, B cells, and mast cells were found in the areas of increased sensitivity [17].

## Discussion

A site-specific neuro-inflammatory response is probably the trigger for vulvodynia. Heparanase discharged from mast cells degrades connective tissue and epithelial basement membranes, allowing proliferating nerve fibers to penetrate the epithelium [18]. This intraepithelial hyperinnervation and the local neuro-inflammatory response [19] results in the local hyperalgesia and allodynia that are characteristic of vulvodynia.

**Fig. 3:**
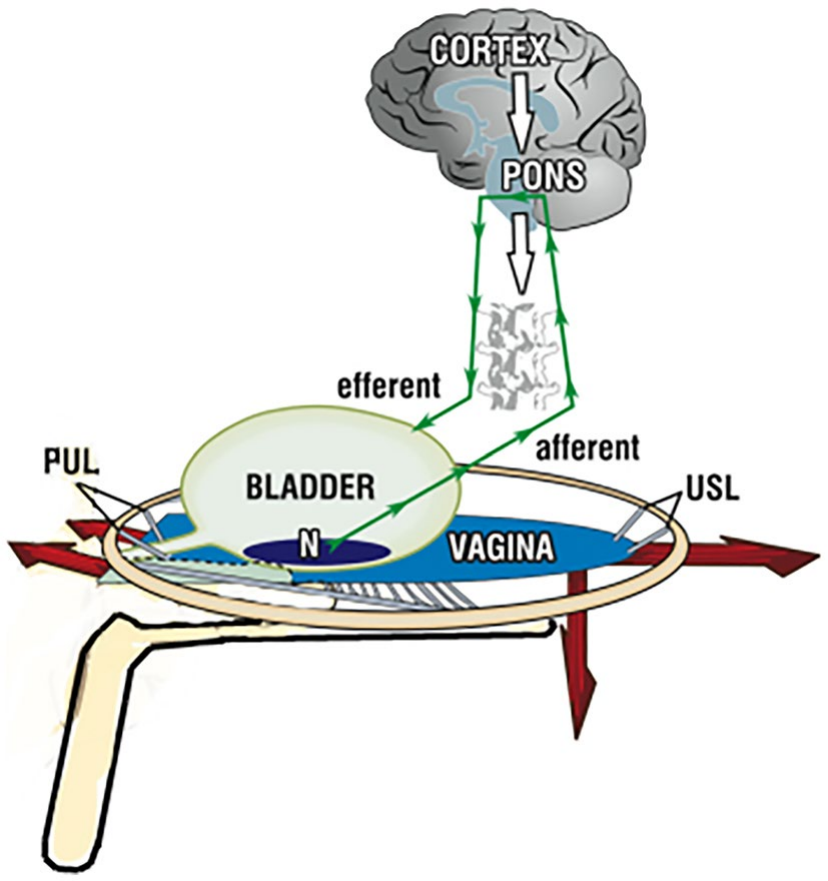
Speculum test: supporting the posterior vaginal fornix with a speculum blade

The theory that USL laxity may be the primary trigger of the local neuro-inflammatory response, by mechanical stimulation of the Frankenhauser ganglia and intrapelvic autonomic sensory plexuses leading to the pain syndrome, has significant therapeutic implications. Pain relief can follow surgical sling reinforcement of USLs [20–22]. This mechanism can be validated by a pre-operative clinical test using intravaginal support with a speculum [23] (Fig. 3), by supporting the posterior fornix with a large intravaginal swab [7], or local anesthetic infiltration of the anatomical site of the visceral plexuses [8, 9]. In a positive speculum test, withdrawal of the supporting speculum is followed by immediate return of the pain and, if present, urinary urgency. In about half of women with vulvodynia, increased vestibular sensitivity is found only in its posterior half [2]. Others have both anterior and posterior allodynia. Damage to the T11–L2 visceral sympathetic sensory plexus may cause referred pain in the upper part of the vagina. In contrast, damage to both sympathetic and parasympathetic components will potentially cause pain in both the upper and lower vagina, since S2–4 spinal root distributions are involved. The vulvodynia seen in general gynecology is often associated with other sites of pelvic pain. Bladder and, sometimes, bowel symptoms occur as part of the “Posterior Fornix Syndrome,” which is caused by lax uterosacral ligaments and cured or improved by repair [24]. However, about half of women with provoked vulvodynia are primary; that is, it started from the first experience of intercourse. This suggests that hyperinnervation may sometimes be constitutional, perhaps arising from a congenital USL laxity, causing similar activation of the visceral nerves and a consequent local neuro-inflammatory response.

The neuro-inflammatory mechanism for vulvodynia raises questions about possible neuro-inflammation in other chronic pelvic pain disorders. Neuro-inflammatory pathology of the bladder wall in interstitial cystitis, with a prominent mast cell response, is well documented [25]. Neuro-inflammation is a physiological process in which inflammatory mediators, especially active peptides, such as substance P and calcitonin gene-related peptide (CGRP), are released from fine unmyelinated afferent somatic C-fibers in response to low-intensity mechanical or chemical stimulation [19, 26]. These peptides trigger a series of inflammatory responses that may include a mast cell and leukocyte response in the affected tissue [27], with proliferation of fine sensory axons. The latter can cause hyperalgesia and allodynia. Relief of vulvodynia by repair of the USL implies resolution of the characteristic histopathological findings. However, no prospective study of premenopausal women with symptomatic vulvodynia, which would involve vestibular biopsies to document histological neuroproliferative changes and their resolution after USL sling repair, has yet been undertaken. Such a study would be difficult to justify. Since the procedure produces a cure, this should, therefore, be sufficient to validate the mechanism we describe. At present, there is no satisfactory non-invasive method, such as immunosuppressive medication, for managing the neuro-inflammation.

## Contributions to existing knowledge

Since provoked vulvodynia can, in some cases, be reduced by applying mechanical support to the posterior vaginal fornix, either by the speculum test or by a broad Q tip swab, it indicates that the source of the pain may be in the uterosacral support system for the visceral nerve plexuses. This treatable cause of vulvodynia possibly accounts for other chronic pelvic pain disorders. However, we cannot yet claim that this is the only cause of vulvodynia or chronic pelvic pain of uncertain cause until there is greater experience with USL slingplasty in the management of these pain disorders.
